# Simple Technique for Microscopic Evaluation of Active Cellular Invasion into 3D Hydrogel Constructs

**DOI:** 10.1021/acsbiomaterials.2c01015

**Published:** 2023-02-07

**Authors:** Christopher R. Simpson, Brenton L. Cavanagh, Helena M. Kelly, Ciara M. Murphy

**Affiliations:** † Tissue Engineering Research Group, Department of Anatomy & Regenerative Medicine, Royal College of Surgeons in Ireland (RCSI), 123 St. Stephen’s Green, Dublin D02 YN77, Ireland; ‡ Cellular and Molecular Imaging Core, Royal College of Surgeons in Ireland (RCSI), 123 St. Stephen’s Green, Dublin D02 YN77, Ireland; § School of Pharmacy and Biomolecular Sciences, RCSI, Ardilaun House, 111 St Stephen’s Green, Dublin D02 VN51, Ireland; ∥ Advanced Materials and Bioengineering Research (AMBER) Centre, Naughton Institute, Trinity College Dublin (TCD), Dublin D02 PN40, Ireland; ⊥ Trinity Centre for Biomedical Engineering, Trinity College Dublin, 152-160 Pearse Street, Dublin D02 R590, Ireland

**Keywords:** hydrogel, migration, invasion, 3D printing, biomaterials

## Abstract

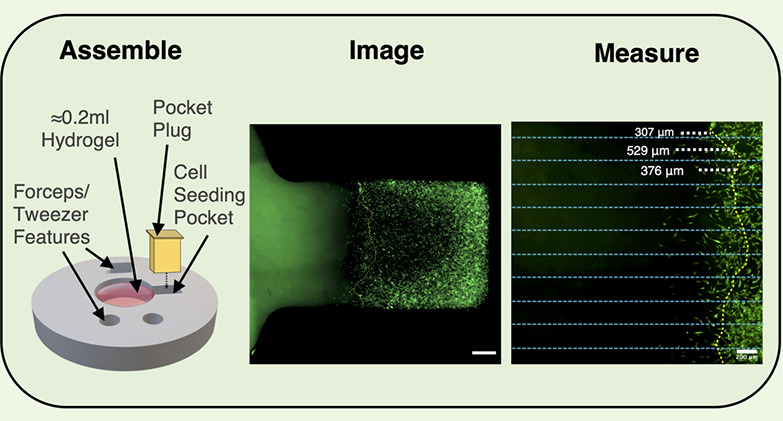

Materials that are evaluated for bioengineering purposes are carefully tested to evaluate cellular interactions with respect to biocompatibility and in some cases cell differentiation. A key perspective that is often considered is the ability for decellularized synthetic or natural based matrices to facilitate cell migration or tissue ingrowth. Current methods of measuring cell migration range from simple scratch assays to Boyden chamber inserts and fluorescent imaging of seeded spheroids. Many of these methods require tissue processing for histological analysis and fixing and staining for imaging, which can be difficult and dependent on the stability of the hydrogel subject. Herein we present a simple platform that can be manufactured using 3D printing and easily applied to in vitro cell culture, allowing the researcher to image live cellular migration into a cellular materials. We found this to be an adaptable, cheap, and replicable technique to evaluate cellular interaction that has applications in the research and development of hydrogels for tissue engineering purposes.

## Introduction

Hydrogels have become a major category of biomaterials in the field of tissue engineering. They have well established characterization protocols,^1,2^ provide a suitable matrix for extracellular architecture^3^ and nutrient diffusion,^4^ and can be tuned to possess appropriate characteristics for a desired application. These matrices offer the ability to deliver cells, environmental cues, or signaling molecules to influence cell behavior.^3^ Crucially, the use of acellular hydrogels are an attractive solution to overcome the translational barriers associated with inclusion of autologous or allogenic derived cells.^5−7^ With such acellular biomaterials there is a key reliance on the host, site-specific cells to appropriately interact with that material through initial migration, followed by deposition of the native tissue matrix.^8^ Hence, evaluating the capacity of acellular hydrogel materials to allow or encourage cell in-migration is frequently demonstrated as an important process preceding effective tissue regeneration. The most commonly used approaches to assess cell movement within a biomaterial include histological sectioning and staining,^9^ Boyden chamber inserts,^10,11^ and migratory scratch assays.^12,13^

**Figure 1:**
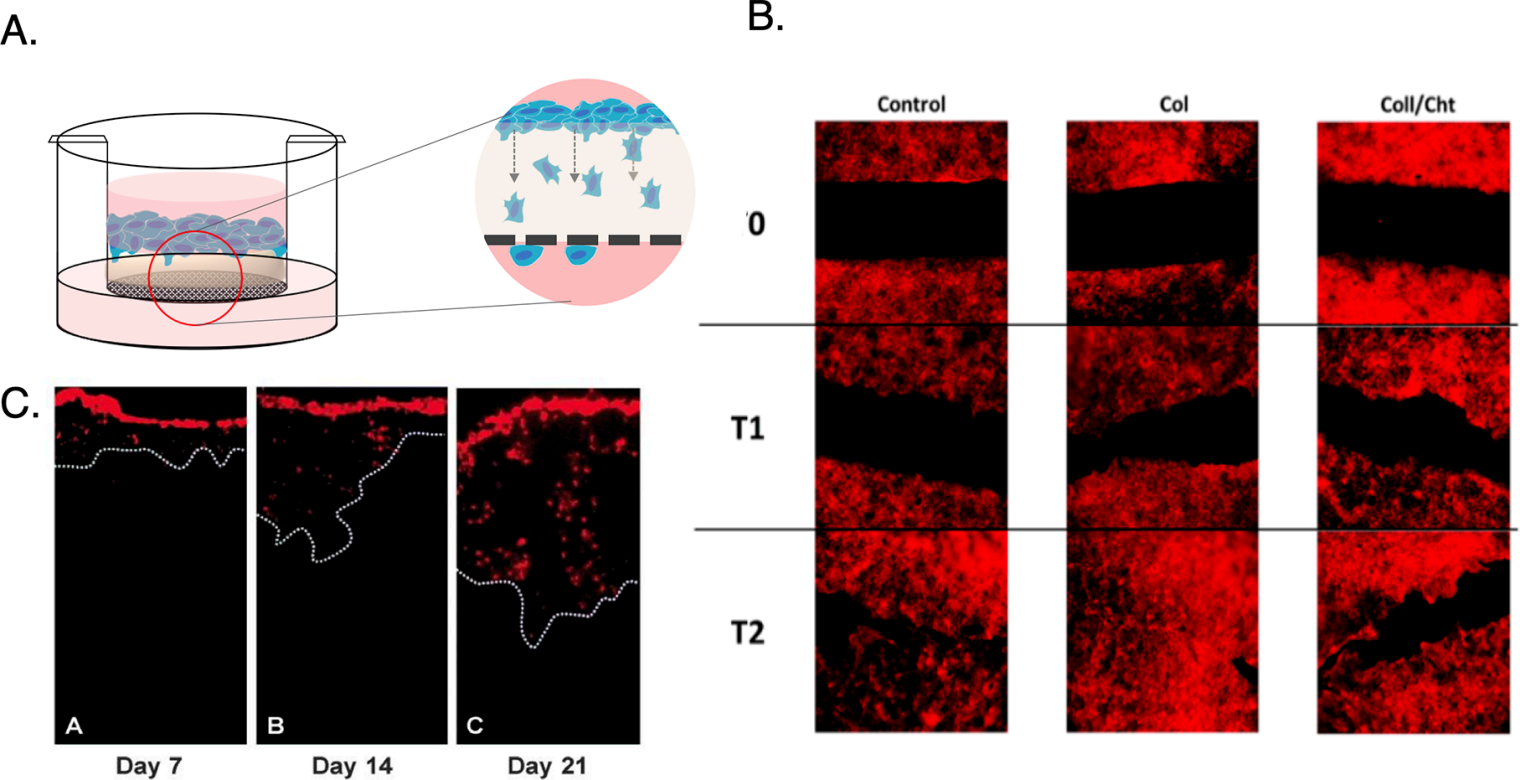
Various methods have been developed to evaluate cell migration with acellular materials. (A) Boyden Chamber set ups quantify the number of cells that migrate through the hydrogel and through the membrane. (B) Scratch assays have been modified to observe motility of cells into a void filled with biomaterial. Reproduced with permission from ref (17). Copyright 2010 John Wiley and Sons. (C) Histological sectioning and staining can show cells within a matrix provided the material is compatible with the processing. Figure reproduced with permission from ref (19). Thesis authored by Rita. I. R. Ibanez, Royal College of Surgeons in Ireland.

Histological processing is a staple when assessing tissue morphology and compositions. The potential to view multiple, discrete tissue strata and surrounding matrix composition allows assessment of material and cell interaction and behavior. These techniques have been adapted and applied to a plethora of different solid scaffold materials such as alginate,^14^ collagen,^15−17^ and fibrin.^18^Figure 1C illustrates the use of cryosectioning and subsequent fluorescent staining of a type 1 collagen hydrogel, to effectively observe and measure infiltration of rat bone-marrow derived mesenchymal stem cells.^19^ However, the success of fixing, embedding, and sectioning of certain hydrogels is highly dependent on the hydrogel matrix material and its intrinsic chemistry with tissue processing techniques. This can make it impossible or laborious to achieve appropriate sectioning and staining that conserves the morphology of these matrices.^9^ Moreover, histological processing is an end point analysis technique and so observed migratory distances over time is a nonmatched measurement.

To circumvent the requirement for histological processing, approaches that do not require serial fixation, tissue processing, and staining are employed. One such procedure utilizes Boyden chamber inserts adapted for assessing the cell mobility into hydrogel materials.^20^Figure 1A outlines the typical setup of this technique; a small amount of hydrogel is applied into the inset and cells are seeded on top.^10^ A chemoattractant is typically applied to media in the lower well and the cells will initiate migration up the chemoattractant gradient. At each time point the hydrogel and uninvaded cells can be swabbed from the chamber, the trans-well membrane removed, and the migrated cells on the bottom of the membrane can be stained or counted.^21^ This technique been successfully applied to assess the invasiveness of certain cell types, the increased motility of cells in response to different ligand inclusions,^22,23^ and cell migration responses to matrix mechanical strength.^24^ This approach is an absolute assessment of migration and will only show the number cells that have migrated through the hydrogel; active cell migration or cells that have adhered to the internal hydrogel matrix are removed with the hydrogel in order to stain the well-insert membrane.

To address this limitation, some experiments utilize fluorescently labeled cells and subsequently image via confocal microscopy.^24^ Migration depth can be determined by stacking images and measuring *z*-axis focal distance. This technique is also limited by the requirement of transferring the chamber insert from the sterile culture environment for imaging as well as the required use of fixatives and staining. In addition, the use of stains, chemo attractants, and individual well-inserts at each time point can be very costly.

Similarly, assessing migration of cells from spheroid cultures using confocal and phase contrast microscopy is a well reported technique. The measurement of distance is conducted in a similar fashion to our developed platform, in that an initial boundary is defined and the changes in area or distance moved are recorded. Ho et al. use GFP human MSCs spheroids to assess migration in response to density of RGD ligand functionalized within an alginate hydrogel.^25^ Similarly Valdoz et al. report the development of a hydrogel dish to accommodate multiple spheroid containing alginate hydrogels, and report normalized changes in area of the spheroids in response to epithelial-to-mesenchymal transition inhibitors.^26^ However, the use of spheroid cultures generally involves seeding cells within the hydrogel and assessing migration outward from within the hydrogel matrix. They are not often appropriate for assessing cellular migration into an acellular hydrogel.^27^

Scratch assays are a simple and inexpensive motility assessment of cells growing back into an open space in a monolayer. The void in the cell layer can be created by culturing cells around a spacer and subsequently removing the spacer from the culture surface, or by culturing a fully confluent monolayer and then mechanically removing a section of the layer creating a void by scratching. The cells that are remaining can then be imaged as they move into the created gap. The scratch assay is frequently used in assessing therapeutic impacts in cell motility or wound healing capability.^28,29^ With respect to tissue engineering, scratch assays appear as an adapted assay to determine hydrogels’ and solid biomaterials’ capacity for cell invasion and interaction. Figure 1B demonstrates the use of a collagen-chitosan hydrogel enabling epithelial cells repopulate a void created in a confluent monolayer. The re-establishment of a confluent layer indicated the capacity of the developed materials to improve the intergradation of adjacent cells, suggesting a potentially positive effect on wound healing.^13^ While it is a cheap and easy assay, the setup can be time-consuming as a cell monolayer is required to be cultured first. A further limitation is the inconsistency of creating the void and this can lead to challenges defining measured distances of migration. Hence, in some cases the area covered is reported. Crucially, unless confocal microscopy is used, the cellular movement is only observed on the surfaces of the subject biomaterial and understanding the movement of cells into the materials is limited.

To overcome the limitations of these well established methods and investigate active cell penetration and migration into an acellular hydrogel matrix, we have developed a simple, cheap and effective alternative technique for *in vitro* use.. This cell migration platform consists of a removable, 3D printed mold with an adhered bottom coverslip. After application of the hydrogel, GFP or fluorescently tagged cells can be seeded adjacently and lateral uniaxial cell migration can be observed over time. This *in vitro* platform is able to accommodate a range of cross-linking methods, does not involve chemical fixation, staining or tissue processing and can observe viable migrating cells within the matrix. Additionally, a fluorescent microscope that has an incubator sample platform can image via a live time-lapse or the same constructs captured at different time points. In this protocol the computer-aided design (CAD) file required to print the molds is provided and their subsequent assembly is described. The steps then required to set up almost any sol–gel transitioning hydrogel or state transitioning material to observe cell migration are outlined.

## Materials and Equipment

### Reagents

- Dulbecco’s Modified Eagle Medium (DMEM) (4500 mg/L glucose, without l-glutamine, without sodium pyruvate)
- Foetal Bovine Serum (FBS)
- Primocin (Antibiotic, InvivoGen)
- MEM Non-Essential Amino Acids Solution (100×) (Gibco)
- GlutaMAX Supplement (100×) (Gibco)
- Dulbecco’s Phosphate Buffered Saline (PBS) (modified, without calcium chloride and magnesium chloride, sterile) (Sigma-Aldrich)
- Soft Paraffin (Vaseline)
- Hard Paraffin
- 70% Ethanol
- Hydrogel (thermoresponsive methylcellulose-collagen 0.1–0.4% w/v)
- Green Fluorescent Protein Rat Mesenchymal Stem Cells (Lonza, Biosciences)

### Equipment

- Micro/Needle files
- Flat end 5 mm spatula
- Glass coverslips 32 mm
- 1 mL Eppendorf Tube Lids
- Forceps/Tweezers
- Hot Plate
- Ultimaker (3D Printer)
- Polylactic acid filament (Ultimaker Tough PLA, Black)
- OnShape Free (Online CAD Software, https://www.onshape.com/en/products/free)
- Ultimaker Cura Slicing Software (Version 4.0)
- Cell culture hood
- UV lamp
- UV blue light (405 nm)
- Oven
- Sterile 6-Well Cell Culture Plates
- Micropipettes (10–1000 μL)
- Pipette gun
- Water bath
- Incubator
- Cell culture flasks
- Inverted fluorescent microscope with controllable stage (Celldiscoverer 7, Carl Zeiss Ltd., Cambridge, UK)
- FIJI Software (Version 2.0.0-rc-69/1.52p)^30^
- Dotted Line ImageJ Plugin (https://imagej.nih.gov/ij/plugins/dotted-line.html)

### Overview of Fabrication, Assembly, and Application of Migration Platform

1. 3D print molds and prepare for assembly
2. Apply glass coverslip and melt on with wax adhesive
3. Prepare GFP cells by FBS starvation overnight
4. Sterilize constructs
5. Apply and cross-link hydrogel
  a. Thermoresponsive cross-linking or
  b. UV Photo-cross-linking
6. Swell hydrogel with complete media and rinse excess
7. Seed cells to adjacent pocket and incubate
8. Image constructs using a fluorescent microscope
9. Image analysis

#### 1 3D Print Molds and Prepare for Assembly

**Figure 2:**
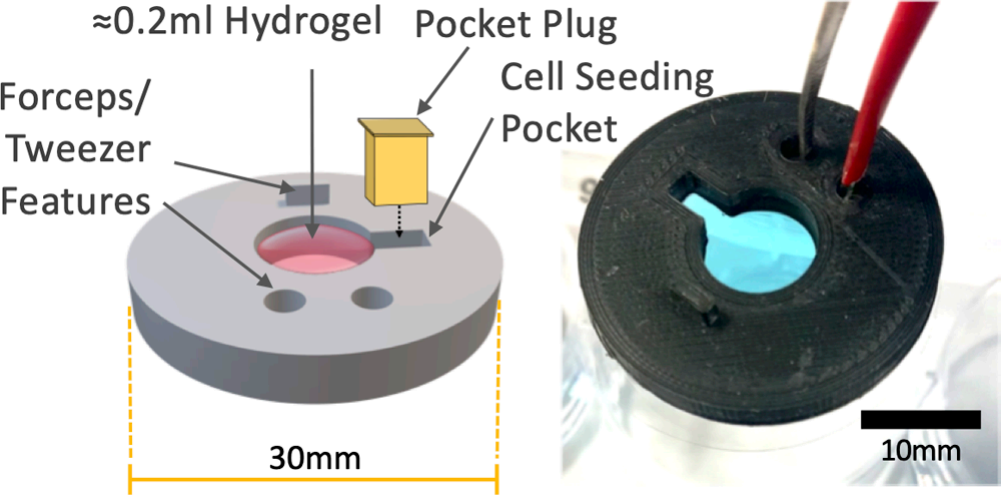
Design of 3D assembly includes a cell seeding pocket, pocket plug to prevent hydrogel leakage into seeding pocket, glass coverslip base for imaging, and forceps and tweezer features for manipulation.

Figure 2 outlines the schematic for the 3D printed construct (30 mm diameter) optimized to fit 6 well culture plates, designed using OnShape Free online CAD software. The STL file for the mold is available at the CAD site via the link at the end of the paper. The CAD file was sliced using Ultimaker Cura (v4.0) and the model was printed using an Ultimaker S5 3D printer extruding tough black PLA (Ultimaker). See the Supporting Information for the complete 3D printing parameters. The molds were printed with a skirt to prevent warping or lifting from the printing bed. At the end of the print, excess skirting was removed; a needle file can be used to smooth out remaining small parts of the skirting.

#### 2 Apply Glass Coverslip and Melt on with Adhesive

An untreated glass coverslip was attached to the underside of the mold to prevent leakage of hydrogel, cells and media. Adhesion of the glass coverslip was achieved by carefully using a 5 mm spatula to spread a thin layer of molten 3:1 hard to soft paraffin around the central cavity edges.

**Note:** Attention should be focused on the edges of the cell seeding pocket; a complete seal is necessary to avoid leakage of cells under the mold.

The molds were then set paraffin side up. A 32 mm glass coverslip was placed on the applied paraffin before heating to 75 °C for 20 min. This inversion and heating allows adhesion and sealing of the glass coverslip without applying pressure.

**Figure 3:**
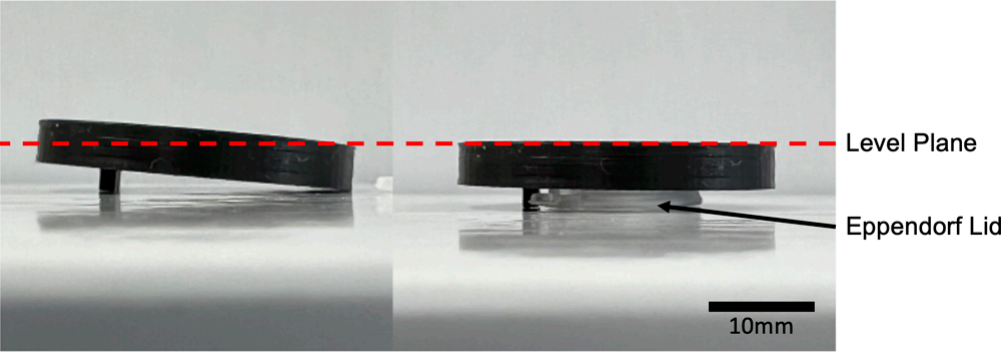
Use of an Eppendorf lid can level the molds to allow optimal application of glass coverslips and even melting.

**Note:** A severed Eppendorf lid can be fitted into the central cavity to stabilize and level the mold when upside-down (Figure 3).

#### 3 Sterilise Constructs

**Figure 4:**
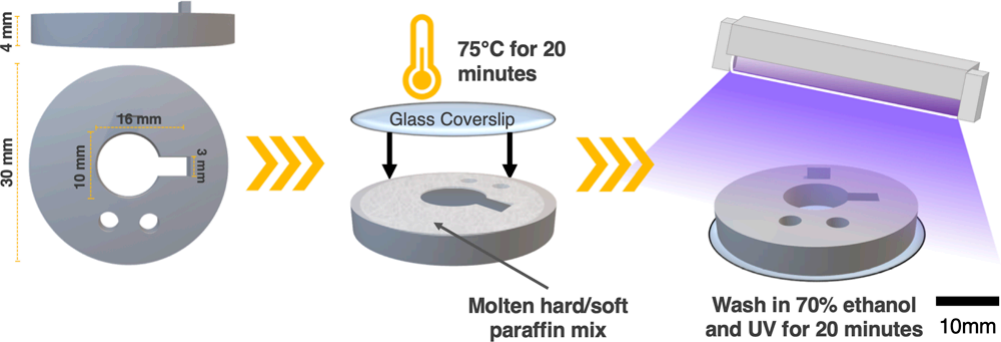
Molds were 3D printed PLA designs as shown. Molten 3:1 hard:soft paraffin was spread along the bottom surface before centering a 32 mm glass coverslip. The constructs were then reheated to 75 °C to remelt the paraffin and seal the edges of the central cavity. Sterilization was achieved via 70% ethanol washing, air drying in a cell culture hood, and finally UV light exposure for 20 min.

**Note:** Residual ethanol will cause the coverslip to adhere to the bottom of the well plate and possibly detach on manipulation.

The mold and coverslip assemblies were sterilized by initial soaking in 70% ethanol. In a cell culture laminar flow hood, using sterile tweezers, molds were then removed from ethanol and placed at an angle in individual wells of a 6-well plate and left to allow complete evaporation of ethanol. After evaporation the molds were then placed flat in the wells and exposed to UV for 20 min (Figure 4).

#### 4 Prepare GFP Cells and FBS Starve Overnight

GFP rMSCs (Lonza Biosciences) were expanded in complete DMEM consisting of 10% FBS, 1% GlutaMAX, 1% MEM NEAA, and 0.2% Primocin. Cells were passaged, using trypsin, at 80–90% confluency and experimentally seeded at passage 7. Prior to seeding GFP rMSCs were serum starved overnight in completed DMEM with a reduced 1% FBS content. All cell culture plates were incubated at 37 °C, 5% CO_2_ in a humidified atmosphere.

#### 5 Apply and Cross-Link Hydrogel

##### a Thermoresponsive Cross-Linking

**Figure 5:**
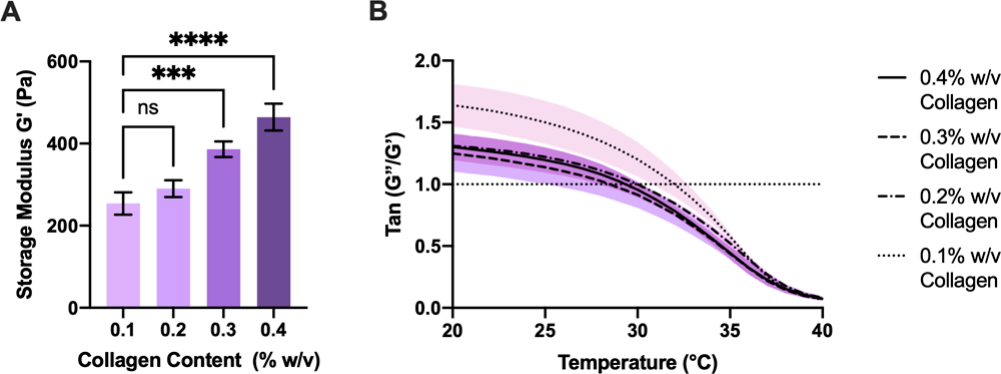
Oscillatory rheological data (A) showing increases in mean storage modulus (+-SD) with increasing collagen content achieved after 25 min incubation at 37 °C. (B) Thermoresponse of methylcellulose-collagen hydrogels was determined as when mean *G*′′/*G*′ = 1. Gelation occurred between 29.2 and 31.8 °C. All rheological tests were conducted with an oscillatory test at 1% strain and a frequency of 1 Hz using a cone (4°) and plate geometry. Data plotted is mean ±SD.

In our exemplar study, 200 μL of a methylcellulose based thermoresponsive hydrogel with increasing amounts of collagen (0.1–0.4% w/v) was used as a validation tool in order to determine whether our newly developed system can detect differences in cell migration in hydrogels with varying composition, stiffness, and gelation time. Figure 5 demonstrates the stiffness of gels after 25 min incubation (Figure 5A) and sol–gel transition temperature (Figure 5B) as a result of varying collagen composition. Each hydrogel formulation was pipetted into the central cavity and incubated at 37 °C for 25 min to allow complete gelation

##### b Photo Cross-Linking

**Figure 6:**
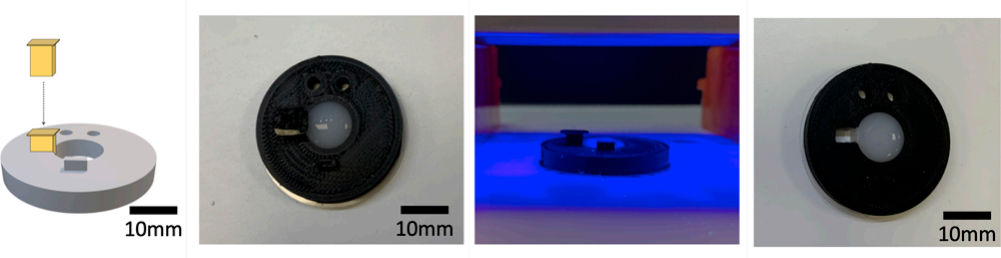
Application of 3D printed plug enabled MeHA-collagen hydrogels to be photo-cross-linked without leaking into the cell seeding pocket.

**Note:** Any cross-linking method could be used at this point; however, the PLA of the 3D printed mold may discolour and breakdown with excess exposure to UV. Photo-cross-linking driven by UV light is feasible with short exposure times.

We further demonstrate the versatility of this platform by using a photo cross-linking hydrogel consisting of a methacrylated-hyaluronic acid collagen (MeHA) hydrogel. Prior to cross-linking, the low viscosity and flowability of the MeHA hydrogel warranted the cell seeding pocket to be blocked by a removable 3D printed plug as shown in Figure 6. The STL file is available via the CAD site at the link given a the end of the paper and the printing parameters used are available in the Supporting Information. Application of 200 μL of hydrogel to the central cavity was held in place by the plug insert; then blue UV light (405 nm) initiated cross-linking for 2 min. After this, the plug was removed using forceps.

#### 6 Swell Hydrogel with Complete Media and Rinse Excess

200 μL of complete DMEM (10% FBS) was applied to the cell seeding pocket and allowed to cover the entire surface of the hydrogel. This was incubated at 37 °C for a further 1 h to allow the hydrogel to swell with FBS containing media. The media was removed and the hydrogels were carefully rinsed twice with prewarmed PBS.

**Note:** Materials should be allowed to reach a swelling equilibrium to avoid changes in the defined boundary. Therefore, as a caveat, materials that significantly swell or shrink may not be appropriate for this platform.

#### 7 Seed Cells to Adjacent Pocket and Incubate

75 μL of DMEM (1% FBS) containing 20 000 serum starved GFP rMSCs were seeded into the cell seeding pocket. Cell seeded hydrogel-mold constructs were immediately incubated at 37 °C, 5% CO_2_ in a humidified atmosphere. Media in the cell seeding pocket was changed every day with 100 μL of fresh DMEM (1% FBS) to avoid dehydration.

#### 8 Image Constructs Using a Fluorescent Microscope

**Figure 7:**
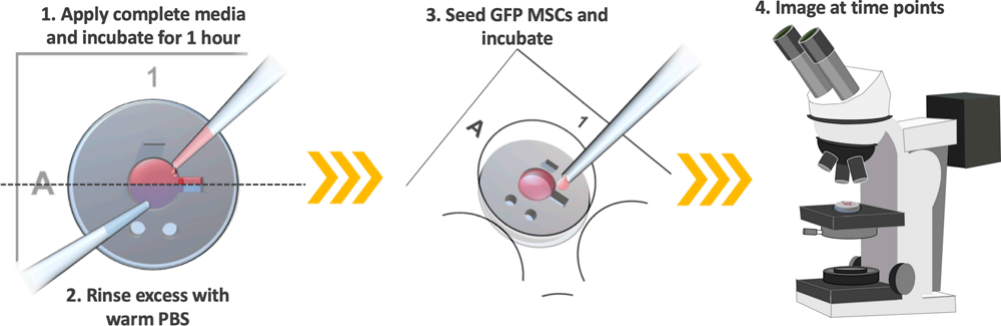
Hydrogel materials can be applied to the central cavity and cross-linking can be initiated. Once cross-linking is achieved, the hydrogel can be soaked with complete growth media for 1 h to enable internal absorption of FBS as a chemoattractant. Excess media can be gently rinsed off with PBS. GFP of fluorescently tagged cells can then be seeded in the cell seeding pocket. At desired time intervals, the entire plate or individual molds can be imaged.

At days 1, 3, and 7, each of the constructs was imaged on the plate, using a Celldiscoverer 7 fitted with an Axiocam 506 camera, Plan-Apochromat 5× (0.3 N.A.) objective and 0.5× optovar (Carl Zeiss Ltd., Cambridge, UK). Higher magnification is possible provided the insets are removed from the well plates. This would end the experimental run, and in the case of our exemplar study, we opted to keep the insets within the plate to image the complete time series. See Figure 7 for a schematic of steps 6–8.

Tiled phase gradient contrast and fluorescent images covering the entirety of the construct were captured using an excitation wavelength of 470 nm and an emission wavelength range of 501–547 nm. Tiled images were then stitched using the stitching module of the ZEN 3.1 (blue edition).

#### 9 Image Analysis

**Figure 8:**
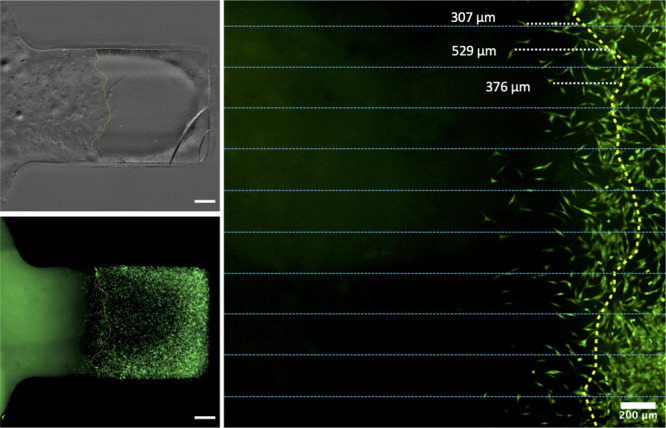
Example contrast and fluorescent microscopy images captured at day 3. Swelling from media treatments can be observed from the intrusion of the hydrogel into the seeding pockets. However, the boundary defined at day 1 did not change by swelling or shrinkage and therefore the day 1 boundary was used over subsequent observations at days 3 and 7. A dotted line was overlaid on the hydrogel boundary and the perpendicular distance from the center of the furthest cell was measured in 10 segments across the hydrogel boundary. Scale bars on left images = 500 μm.

Measurement of migration distance was measured using FIJI software. Each construct was individually opened and rotated (transposed) to the same orientation with the cell seeding pocket at the 3 o’clock position. At day 1, the hydrogel boundary was defined using the phase contrast images and coupled with observation of GFP cell distribution (Figure 8). The boundary was defined using the segmented line selection tool and saved using the ROI manager for reference at later time points. A dotted line plugin was used to overlay a dotted line on the segmented selection.

The seeding pocket was divided into 10 segments as shown in Figure 8 and the distance migrated by the furthest cell in each segment was measured. Specifically, the maximum distance migrated was defined as the perpendicular distance from the hydrogel boundary to the closest end of the cell.

## Anticipated Results

**Figure 9:**
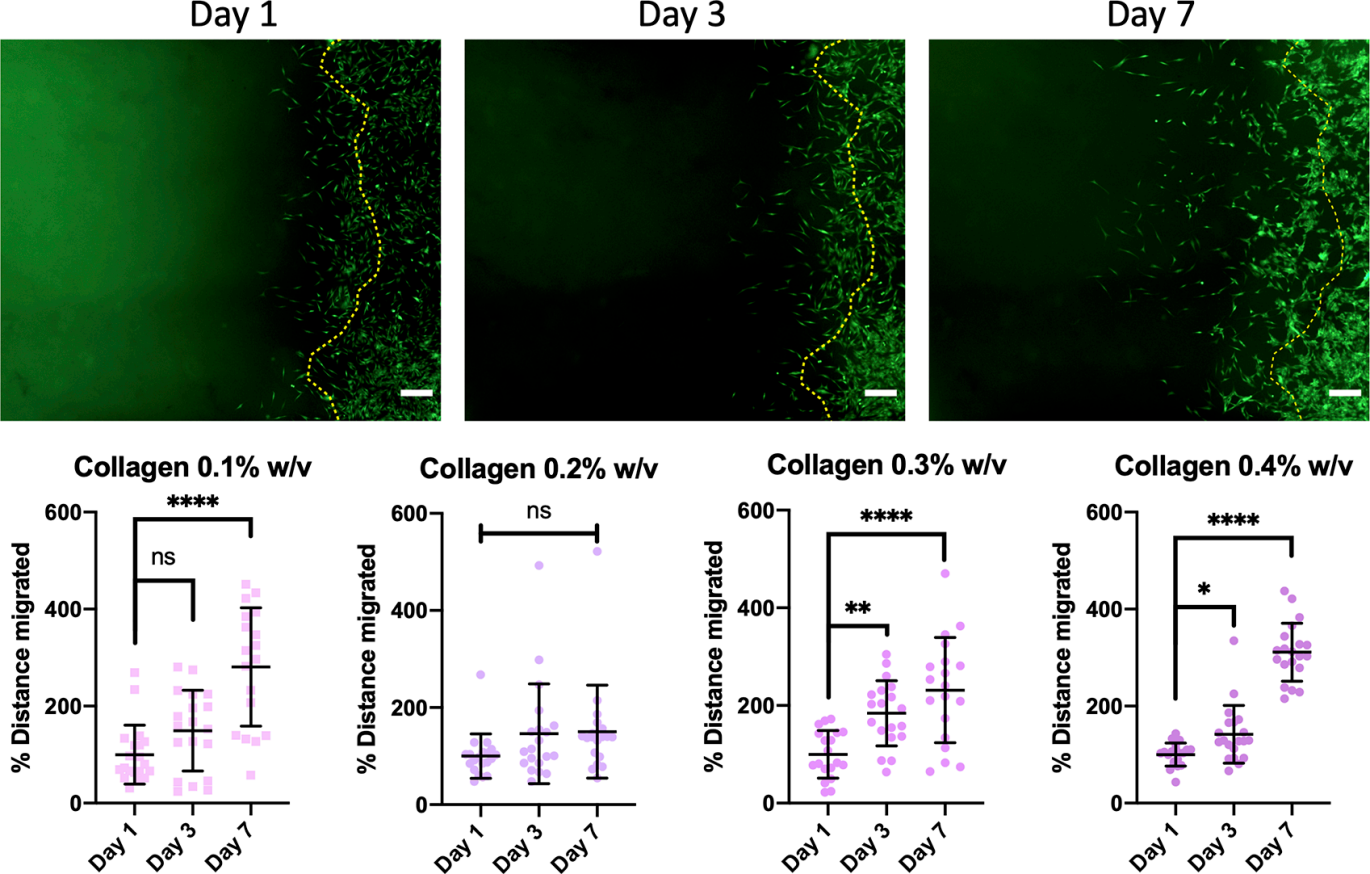
Cells were shown to increase in their distance from the edge of the hydrogel boundary over time. With increasing the collagen content of a thermoresponsive methylcellulose, it was observed that greater significant increases in the distance GFP rMSCs in-migrate were found in the highest collagen content (0.4% w/v) group. Mean migration distance as a percentage of distance covered at day 1 are shown ± SD from two independent experimental runs (scale bar = 200 μm).

**Figure 10:**
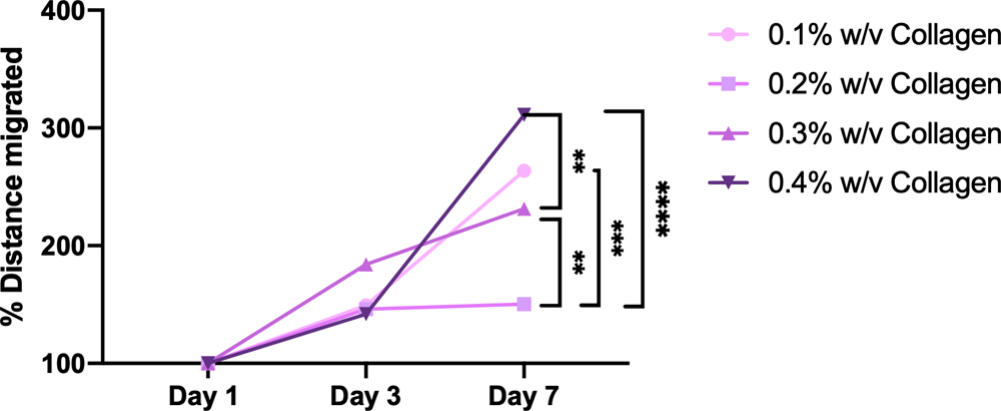
Mean distance increases (%) from day one illustrate the infiltration observed over the 7 days.

An example of the results obtained using this 3D printed migratory platform is shown in Figure 9, comparing the migration of GFP rMSCs in response to increasing the collagen content of a thermoresponsive hydrogel system. The migration platform with the hydrogel was successfully used to measure cell migration into an acellular matrix over 7 days. At each time point a sample of migrated cells could be measured from the hydrogel boundary. The images captured show significant increases in migration distance of the cells within each hydrogel between days 1 and 7, with the exception of 0.2% w/v collagen where no significant increases in migration distance were observed over time. Crucially, the largest and most consistent increases in cell migration over time were observed with the highest collagen content of 0.4% w/v collagen (Figure 10). There was a significant increase in cell migration with increasing collagen content between the 0.2–0.4% w/v collagen groups. While increasing collagen content is hypothesized to provide increasing ligand availability, interestingly, the percent distance migrated in the 0.1% w/v collagen group between days 3 and 7 was significantly higher than that for the 0.2%w/v collagen group, suggesting other factors are at play. These anticipated results demonstrate the value of this system as a new means to assess active cell migration into suitable hydrogel environments. However, this platform is not intended as a stand-alone technique for hydrogel characterization, but an important tool to be used in conjunction with thorough physicochemical characterization to fully elucidate the behavior and performance of hydrogel based systems.

## Discussion

Acellular hydrogel biomaterials for tissue regeneration have become an attractive option for readily available implants to drive endogenous repair mechanisms. They have been researched for multiple clinical applications including skin,^5^ intervertebral disc,^31^ cartilage,^32^ bone,^33,34^ and cardiac repair.^6^ Crucially, cells local to the site of implantation must sufficiently in-migrate to appropriately initiate repair and eventual integration of these materials. We present a cheap and effective way to demonstrate and quantify migration of MSCs into an acellular hydrogel. This protocol circumvents the need for lengthy, often matrix compromising fixing, embedding, and sectioning required with histology while offering the advantage, over Boyden chamber set-ups, by quantifying the degree of migration.

A simple 3D model was created to facilitate a central cavity to house up to 200 μL of hydrogel, an adjacent cell seeding pocket and gripping features for easy manipulation of the platform on the plate. The design was printed using a tough PLA polymer from an Ultimaker 3D printer and then completed by adhering a glass coverslip to the underside of the design using a blend of soft and hard paraffin (1:3). The entirety of the assembled platform was sterilizable with 70% ethanol and brief UV exposure of 15 min. Moreover, the CAD model can be flexibly modified to accommodate a range of gel morphologies, volumes, and swelling capacities in the central cavity, as the entire footprint of the mold is covered by the glass coverslip.

We demonstrate the application of this platform to experimentally assess collagen concentrations for a range of candidate thermoresponsive hydrogels. This showcases the ability to implant a shear thinning hydrogel to the hydrogel cavity, initiate cross-linking, swell with a chemoattractant (FBS), apply fluorescently labeled MSCs, and finally image the entire constructs over a time series. Importantly, the simplicity and versatility of the design would permit application of any void filling biomaterial, chemoattractant, cross-linking technique, and of course cell type.

From use of this platform, we were able to quantify different migration distances in response to collagen concentrations ranging from 0.1–0.4% w/v. GFP rMSCs were found within the hydrogel matrix of all formulations from 24 h. Successive imaging on days 3 and 7 observed further in-migration of MSCs in all formulations. Notably, Figure 10 shows that larger increases in migratory distance of 311% were detected with the highest collagen concentration of 0.4% w/v collagen, while lower increases of 150.3% and 231.4% were found in 0.2 and 0.3% w/v collagen, respectively. The role of collagen for cell interaction, anchorage, motility, and differentiation is widely reported in the literature and exploited to improve biocompatibility of implantable materials.^35,36^ Lutolf et al. reported a biphasic effect of RGD ligand concentration on invasion rate.^37^ They present, an increase of invasion rate with increasing RGD up to 85 μM. Our results similarly show, inclusion of more collagen from 0.2–0.4% w/v in the hydrogel formulation has encouraged more invasion of MSCs into the matrix. However, we measured large increases of 280.6% at day 7 with the 0.1% group and findings reported by Ho et al. also show increased migration with a lower RGD ligand density compared to a higher density, from a spheroid culture. Our results suggest either a biphasic effect of collagen concentration in our hydrogel on migration or, more likely, a physical property of the hydrogels is having an effect.

Figure 5A shows increases in stiffness with increasing collagen contents with 0.1% collagen having the lowest stiffness. Investigations into stiffness as a function of cross-linking density are frequently reported and suggest that highly cross-linked networks inhibit the migration of cells into hydrogels.^38^ This offers one possible physical explanation to the high increase in migration, with lower stiffness there could be less resistance to migration, but as we did not see this trend across all groups, there is scope for an interplay of physical properties such as stiffness and porosity^10^ with adhesion site presence.

There are limitations with the proposed method. Highly swelling and unstable materials will fail to maintain an appropriate interface with the cell seeding pocket, making measurement of cell migration not possible. Similarly, materials with low stiffness or low viscosity after cross-linking may also struggle to keep the seeding pocket clear. Lastly, the movement of cells is quantified as strictly uniaxial movement from the boundary, in reality it is unlikely a linear path was taken by each cell. This limitation could be addressed with a time lapse capture of migration within our platform followed by single cell migration analysis using a manual tracking plugin with FIJI.^39^ Adjacent characterization of the physical and physiochemical hydrogel properties is necessary to contextualize and interpret results. While more peripheral research will be needed to determine the mechanisms driving the shown cellular invasion, the proposed technique successfully generated a quantifiable and relevant trend. However, utilized as an individual aspect of hydrogel characterization, we believe this platform has a place in hydrogel characterization and has the potential to indicate cellular affinity for a tested material. Furthermore, due to its versatility, this technique may have potential utility in applications outside of tissue engineering.

## Summary

We outline the fabrication of this platform, its application and analysis to be cheap and feasible for any laboratories that have a 3D printer and fluorescent microscope. The developed platform was successfully used to measure active migratory distances of MSCs. From these observations we were able to optimize a thermoresponsive hydrogel to encourage cell migration into an acellular environment.

## Data Availability

The 3D CAD file of the migration mold is available to download at https://cad.onshape.com/documents/6a02f10148e3136dba062438/w/bd96b84a293ca0405dc9dec2/e/340e8c0deeac0a8d1681f326. The 3D CAD file of the cell seeding pocket plug is available to download at https://cad.onshape.com/documents/c67643227e296f94bb2c2915/w/2660769038c5d8cf8472bacc/e/b4e31ebc05274f2c0750f086.

## Supporting Information Available

Printing parameters of the migration mold and cell seeding pocket plug for the slicing software (PDF)
