# Supplementary material for: Simple Technique for Microscopic Evaluation of Active Cellular Invasion into 3D Hydrogel Constructs
Source: ACS Biomater Sci Eng. 2023 Feb 7;9(3):1243–50. doi: 10.1021/acsbiomaterials.2c01015 (PMC10015425; doi:10.1021/acsbiomaterials.2c01015)
Supplement: Supplementary file 1 — ab2c01015_si_001.pdf [file ab2c01015_si_001.pdf]

Supporting Information

A Simple Technique for Microscopic Evaluation of Active Cellular Invasion into 3D Hydrogel Constructs

*Christopher R. Simpson[1], Brenton L. Cavanagh[2], Helena M. Kelly[1,3], *Ciara M. Murphy [1,4,5]*

1. Tissue Engineering Research Group, Department of Anatomy & Regenerative Medicine, Royal College of Surgeons in Ireland (RCSI), 123 St. Stephen's Green, Dublin D02 YN77, Ireland

2. Cellular and Molecular Imaging Core, RCSI, 123 St. Stephen's Green, Dublin D02 YN77, Ireland

3. School of Pharmacy and Biomolecular Sciences, RCSI, Ardilaun House, 111 St Stephen's Green, Dublin D02 VN51, Ireland

4. Advanced Materials and Bioengineering Research (AMBER) Centre, Naughton Institute, Trinity College Dublin (TCD), Dublin D02 PN40, Ireland

5. Trinity Centre for Biomedical Engineering, Trinity College Dublin, 152-160 Pearse Street, Dublin D02 R590, Ireland

**Corresponding Author. Email: ciaramurphy@rcsi.com*

Number of pages: 6
Number of tables: 2

Table S1- Migration mould printing parameters for generation of sliced 3D model in Ultimaker Cura

Cell Migration Mould

**Slic3r Software Printing Parameters**	**Setting**
**Quality**	
Layer Height	0.1mm
Initial Layer Height	0.2mm
Line Width	0.35mm
Wall Line Width	0.35mm
Inner Wall Line Width	0.3mm
Outer Wall Line Width	0.35mm
Top/Bottom Line Width	0.35mm
Infill Line Width	0.45mm
Skirt/Brim Line Width	0.35mm
Initial Layer Line Width	120.00%
**Shell**	
Wall Thickness	1.23mm
Wall Line Count	4
Top/Bottom Thickness	1.2mm
Top Thickness	1.2mm
Top Layers	12
Bottom Thickness	1.2mm
Bottom Layers	12
Optimize Wall Printing Order	Yes
Fill Gaps Between Walls	Everywhere
Horizontal Expansion	0.0mm
Enable Ironing	No
**Infill**	
Infill Density	20.00%
Infill Line Distance	2.25mm
Infill Pattern	Concentric
infill Layer Thickness	1
Gradual Infill Steps	0
**Material**	
Printing Temperature	210℃
Printing Temperature Initial Layer	210℃
Initial Printing Temperature	200℃
Final Printing Temperature	195℃
Build Plate Temperature	65℃
Build Plate Temperature Initial Layer	65℃
Enable Retraction	Yes

Retract at Layer Change	No
Retraction Distance	6.5mm
Retraction Speed	25mm/s
**Speed**	
Print Speed	30mm/s
Infill Speed	30mm/s
Wall Speed	27mm/s
Outer Wall Speed	21mm/s
Inner Wall Speed	27mm/s
Top/Bottom Speed	24mm/s
Travel Speed	150mm/s
Initial Layer Speed	14mm/s
Skirt/Brim Speed	14mm/s
Enable Jerk Control	Yes
Enable Acceleration Control	Yes
**Support**	
Generate Support	No
**Build Plate Adhesion**	
Enable Prime Blob	No
Build Plate Adhesion Type	Brim
Brim Line Count	9
Brim Distance	8.0mm
Brim Only on Outside	Yes

Table S2- Cell seeding pocket plug printing parameters for generation of sliced 3D model in Ultimaker Cura

Seeding Pocket Plug

Slic3r Software Printing Parameters	Setting
**Quality**	
Layer Height	0.1mm
Initial Layer Height	0.2mm
Line Width	0.35mm
Wall Line Width	0.35mm
Inner Wall Line Width	0.3mm
Outer Wall Line Width	0.35mm
Top/Bottom Line Width	0.35mm
Infill Line Width	0.45mm
Skirt/Brim Line Width	0.35mm
Initial Layer Line Width	120.00%
**Walls**	

Wall Thickness	1.23mm
Wall Line Count	4
Outer Wall Wipe Distance	0.2mm
Outer Wall Inset	0.0mm
Optimize Wall Printing Order	Yes
Outer Before Inner Walls	No
Alternate Extra Wall	No
Compensate Wall Overlaps	Yes
Compensate Outer Wall Overlaps	Yes
Compensate Inner Wall Overlaps	Yes
Fill Gaps Between Walls	Everywhere
Filter Out tiny Gaps	Yes
Print Thin Walls	No
Horizontal Expansion	0.0mm
Initial Layer Horizontal Expansion	0.0mm
Z Seam Alignment	Sharpest Corner
Seam Corner Preference	Hide Seam
**Top/ Bottom**	
Top Surface Skin Layers	0
Top/Bottom Thickness	1.2mm
Top Thickness	1.2mm
Top Layers	12
Bottom Thickness	1.2mm
Bottom Layers	12
Top/Bottom Pattern	Lines
Bottom Pattern Initial Layer	Lines
Top/Bottom Line Directions	[]
No Skin in Z Gaps	No
Extra Skin Wall Count	1
Enable Ironing	No
Skin Overlap Percentage	10.00%
Skin Overlap	0.0325mm
Skin Removal Width	1.25mm
Top Skin Removal Width	1.25mm
Bottom Skin Removal Width	1.25mm
Skin Expand Distance	1.25mm
Top Skin Expand Distance	1.25mm
Bottom Skin Expand Distance	1.25mm
Maximum Skin Angle for Expansion	90.0°
Minimum Skin Width for Expansion	0.0mm
**Infill**	

Infill Density	20.00%
Infill Line Distance	2.25mm
Infill Pattern	Grid
Connect Infill Lines	Yes
Infill Line Directions	[]
Infill X Offset	0.0mm
Infill Y Offset	0.0mm
Randomize Infill Start	No
Infill Line Multiplier	1
Infill Overlap Percentage	0.00%
Infill Overlap	0
Infill Wipe Distance	0.2mm
Infill Layer Thickness	0.3mm
Gradual Infill Steps	No
Infill Before Walls	No
Minimum infill Area	No
Infill Support	No
**Material**	
Printing Temperature	210℃
Printing Temperature Initial Layer	210℃
Initial Printing Temperature	200℃
Final Printing Temperature	195℃
Build Plate Temperature	65℃
Build Plate Temperature Initial Layer	65℃
Enable Retraction	Yes
Retract at Layer Change	No
Retraction Distance	6.5mm
Retraction Speed	25mm/s
**Speed**	
Print Speed	30mm/s
Infill Speed	30mm/s
Wall Speed	27mm/s
Outer Wall Speed	21mm/s
Inner Wall Speed	27mm/s
Top/Bottom Speed	24mm/s
Travel Speed	150mm/s
Initial Layer Speed	14mm/s
Skirt/Brim Speed	14mm/s
Enable Jerk Control	Yes
Enable Acceleration Control	Yes
**Support**	

Generate Support	No
**Build Plate Adhesion**	
Enable Prime Blob	No
Build Plate Adhesion Type	Brim
Brim Line Count	3
Brim Distance	1.0mm
Brim Only on Outside	Yes
